# Supplementary material for: Broadband physical layer cognitive radio with an integrated photonic processor for blind source separation
Source: Nat Commun. 2023 Feb 27;14:1107. doi: 10.1038/s41467-023-36814-4 (PMC9971366; doi:10.1038/s41467-023-36814-4)
Supplement: Supplementary file 1 — Supplementary information [file 41467_2023_36814_MOESM1_ESM.pdf]

Broadband physical layer cognitive radio with an integrated photonic processor for blind source separation

Supplementary information

Weipeng Zhang, Alexander Tait, Chaoran Huang, Thomas Ferreira de Lima, Simon Bilodeau, Eric C. Blow, Aashu Jha, Bhavin J. Shastri and Paul Prucnal

Supplementary text contents:

1. Discussion on kurtosis and modulation format
2. Influence of MRR filter function on signal bandwidth and distortion
3. BSS demonstration on ultra-wideband signals

1. Discussion on kurtosis and modulation format

BSS is known to be agnostic to signal format because its algorithm does not rely on explicit and prior knowledge about the waveform. As described in the Methods in the main article, we utilised the independent component analysis, which follows the central limit theorem and can find the demixing weights that output a signal with the optimised kurtosis. The central limit theory purports that the mixtures of two independent signals should be more Gaussian in amplitude distribution than the original signals before mixing. The kurtosis is a metric associated with the signal distribution and is calculated by the second and fourth statistic moment of signals using Equation (1) below, where $\bar{s}$ is the mean of the signal.

$$\mathrm{kurtosis}(s(t)) = \frac{E[(s(t)-\bar{s})^4]}{(E[(s(t)-\bar{s})^2])^2} \quad (1)$$

A perfect gaussian signal has a kurtosis of 3. Non-gaussian distributed signals can have a kurtosis either larger than or smaller than 3. Thus, an often-used metric to describe the non-gaussianity is the absolute relative kurtosis (ARK), which is calculated by $\mathrm{ARK} = \mathrm{abs}(\mathrm{kurtosis}(s(t)) - 3)$ for a given signal $s(t)$. The search for optimal demixing weights is done through a constrained Nelder-Mead algorithm. This is a non-gradient-based optimisation algorithm that approaches the maximal ARK (maximal non-gaussianity) by iteratively updating a searching simplex, or in our case, the weights applied onto the on-chip microrings. Hence, our approach does not distinguish signals from their modulation format, either digital or analogue. There is no straightforward mathematical evidence on which modulation format has a larger ARK. Supplementary Fig. 1 below displays some standard signals of both modulation types, which suggests that the digital modulation ones reveal smaller ARKs. For an iterative searching procedure like the Nelder-Mead algorithm that we used in this work, a target search point corresponding to a larger ARK is often associated with a larger gradient in its vicinity. Thus, it requires fewer iterative steps to converge, or yields more accurate weights, leading to cleaner separation of the signals. Therefore, separating digitally modulated signals, as is shown in our manuscript, should be a fair and sometimes more stringent task to examine the performance of this photonic blind source separation.

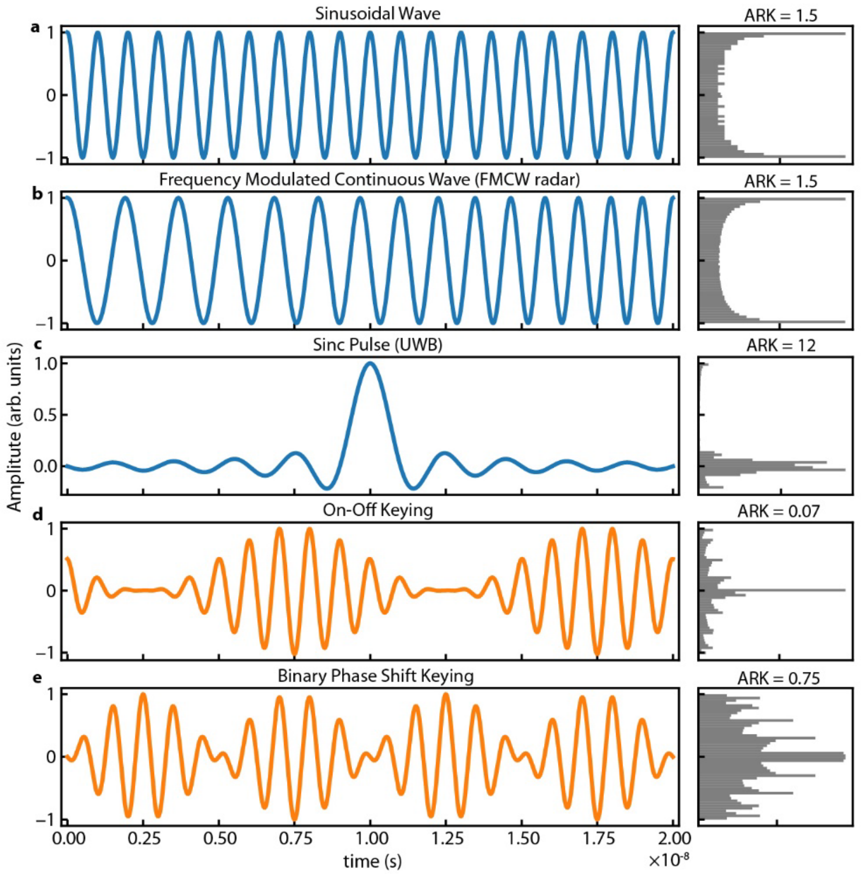

Supplementary Fig. 1. Kurtosis of common analogue and digital modulated signals. These signals share the same carrier frequency of 1 GHz. **a-c** analogue signals that are coloured blue. **d** and **e** digital modulated ones coloured orange. All the corresponded histogram are shown in the right panels.

2. Influence of MRR filter function on signal bandwidth and distortion

Thank you for this constructive comment in terms of bandwidth. Let us first discuss the bandwidth of the MRR filtering function. The resonator-based optical filters, such as the MRRs, achieve weighting of signals by tuning their transmission spectrum (as shown in Fig. 2c and Supplementary Fig. 2 below). Each MRR in the weight bank filters the light from the input port into the thru and drop port, the transmission of the optical power is shown in Supplementary Fig. 2a. Then, a balanced photodetector (BPD) converts the optical power to electrical signals in proportion to the power difference between these two ports, as shown in Supplementary Fig. 2c. In our experiment, we used the two MRRs whose resonance frequencies are centred around 192700GHz and 193200GHz, corresponding to the leftmost and the rightmost peaks, respectively. We used two lasers operating in frequencies within the slopes (the orange and the green regions) and modulated the RF signals onto the lasers. Then, these two MRRs filtered their corresponding laser light to the drop and thru ports, and the resulting electrical output from BPD is the weighted summation of the two input signals. As shown in Supplementary Fig.

2b and d, both positive and negative weights can be applied to carry out the BSS algorithm and suppress the unwanted signal.

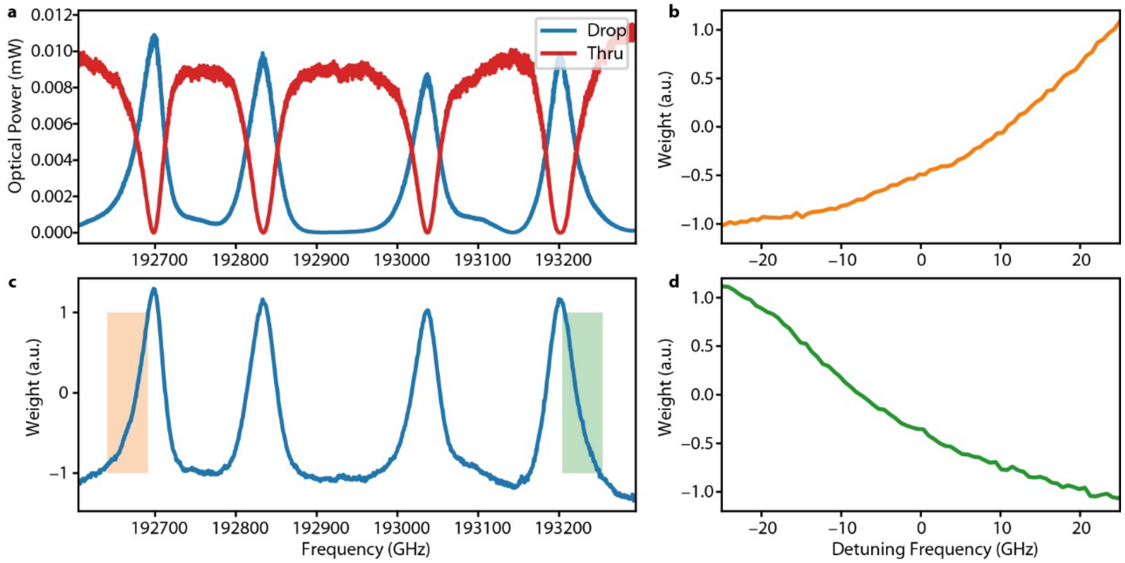

Supplementary Fig. 2. MRR Spectra. a the transmission of the MRR weight bank. The blue and red curves are for the drop and thru ports, respectively. The four transmission peaks and dips correspond to four independent MRRs inside the weight bank, as shown in Fig. 2b. Noting that the y-axis here is in linear scale as opposed to the log scale used by Fig. 2c. **b-d** the weight as a function of the laser frequency. **b** and **d** are the zoomed-in views of the orange and the green regions in **c**, respectively. The curve in **b** is centred at 192666 GHz, and that in **d** is at 193229 GHz.

It is acknowledged that the linewidth of the laser is widened when modulating RF signals onto the lasers. Neglecting the linewidth of the unmodulated laser, the widened linewidth is double the maximal signal frequency, which is approximately the carrier frequency plus half the signal bandwidth. Zooming in on the weighting curves (shown in Supplementary Fig. 2b and c), we can notice that the slope of this profile can theoretically introduce non-constant filtering onto the signal if either the carrier frequency or the instantaneous bandwidth is excessively large. Potentially causing distortions for processed signals, which partially explains why the SIR performance is decreased as the carrier frequency increases, as indicated in Fig. 4. However, the incurred distortions are mitigated by themselves. The transmission profiles of these two MRRs are of very similar Lorentzian shapes because of their almost identical ring radii. As shown in Supplementary Fig. 2b-d, we used different sides of the transmission profiles so that the filtering function from one MRR is positively sloped (Supplementary Fig. 2b) and the other is negatively sloped (Supplementary Fig. 2d). Thus, the uneven frequency responses derived from the slopes are cancelled to some extent when the weighted signals are summed at the BPD, which maintains good suppression of signals with broad bandwidth or large carrier frequencies.

Besides, the slope is associated with the transmission profile width, and intentional narrow widths were designed to avoid spectrum overlaps and the potential crosstalk between

adjacent MRRs. Suppose fewer MRRs are needed, like that only two among the four MRRs were required for the demonstrated BSS experiment, the distortion can be further mitigated by optimising the profile, which is associated with the device geometry, including the radius of the MRRs and the distance between the main and the ring waveguide.

In the main article, we prove this by experiment results of more than 30dB SIR for signals with carrier frequencies of up to 19.2 GHz and bandwidth of 3.2GHz. To further test our claim, we added experimental results below using this photonic BSS system separating two UWB signals (3.1-10.6 GHz). The results prove a similar performance to that shown in Fig. 4, showing SIRs of more than 33 dB. This demonstrated performance is sufficient for most wireless applications that utilize small portions of the spectrum guided by FCC allocation.

2. BSS demonstration on ultra-wideband signals

As discussed above, the signal with a broad instantaneous bandwidth reveals having large distortions being processed with an MRR filter. To prove this, we include another experiment using our photonic BSS to separate two ultra-wideband (UWB) signals from their mixtures. These UWB signals are so broadband that they utilise a band across more than 7 GHz spectrum, as shown in Supplementary Fig. 3 e and f. Besides, these signals appear as analogue-typed modulated pulses, which can speak as an example, proofing that our BSS system is agnostic to the signal format.

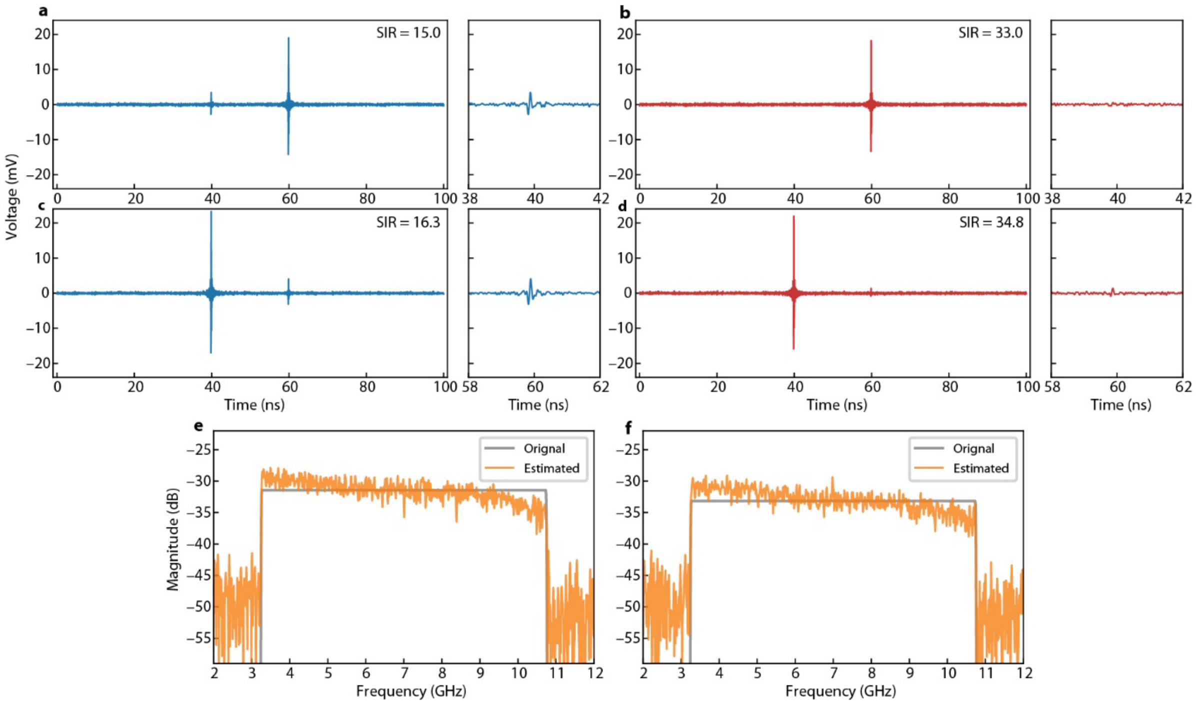

Supplementary Fig. 3. Experiment result on UWB signals. a and **c** the mixtures before BSS. Subplots on the right are the zooming-in view of the mixtures shown on the left. **b** and **d** the estimated signals after BSS. Subplots on the right are zooming-in view of the mixtures shown on the left. **e** and **f** the orange curves are the spectrum of the estimated ones shown in **b** and **d**, respectively. The grey curves are the spectrum of the original sources before mixing and are normalised to have the same power as the estimated signals for the sake of illustrations.

The mixtures shown in Supplementary Fig. 3 a and c are generated using the same mixing matrix as the results shown in Fig. 4, and both have a peak-to-peak amplitude of $1\ \mathrm{V}$. The results in Supplementary Fig. 3 b and d show that the separated signal sources have SIRs of more than 33 dB, delivering consistent good performance. Comparing these two tests (Supplementary Fig. 3 and Fig. 4), the instantaneous bandwidth of the tested signals increases more than two-fold ($\leq 3.2\mathrm{GHz}$ to $\geq 7.5\mathrm{GHz}$), but the separation performance remains the same, which proves our abovementioned conclusions regarding the impact of the MRR transmission profile on signal suppression. As expected in Supplementary Fig. 3 **e** and **f**, the separated source signals are distorted, revealing uneven spectrums after being processed by the MRR weight bank. Given the broad bandwidth of more than 7.5 GHz, this unevenness is reasonably acceptable, with about 5 dB between the maximal and the minimal. Besides, the distortions are primarily in linear shapes, which eases the characterisation and compensation through equalisers.
